# Identification of novel *GNAS* mutations in intramuscular myxoma using next-generation sequencing with single-molecule tagged molecular inversion probes

**DOI:** 10.1186/s13000-019-0787-3

**Published:** 2019-02-08

**Authors:** Elise M. Bekers, Astrid Eijkelenboom, Paul Rombout, Peter van Zwam, Suzanne Mol, Emiel Ruijter, Blanca Scheijen, Uta Flucke

**Affiliations:** 1 Department of Pathology, Radboud University Medical Center, Geert Grooteplein Zuid 10, 6525 GA Nijmegen, The Netherlands; 2 Department of Pathology, The Netherlands Cancer Institute, Amsterdam, The Netherlands; 3 Department of Pathology, PAMM Michelangelolaan 2, 5623 EJ Eindhoven, The Netherlands; 4 Department of Pathology, Jeroen Bosch Hospital, Henri Dunantstraat 1, 5223 GZ Den Bosch, The Netherlands; 5 Department of Pathology, Rijnstate Hospital, Wagnerlaan 55, 6815 AD Arnhem, The Netherlands

**Keywords:** Next generation sequencing, TaqMan genotyping, smMIP assay, *GNAS* mutation, Intramuscular myxoma

## Abstract

**Background:**

Intramuscular myxoma (IM) is a hypocellular benign soft tissue neoplasm characterized by abundant myxoid stroma and occasional hypercellular areas. These tumors can, especially on biopsy material, be difficult to distinguish from low-grade fibromyxoid sarcoma or low-grade myxofibrosarcoma. *GNAS* mutations are frequently involved in IM, in contrast to these other malignant tumors. Therefore, sensitive molecular techniques for detection of *GNAS* aberrations in IM, which frequently yield low amounts of DNA due to poor cellularity, will be beneficial for differential diagnosis.

**Methods:**

In our study, a total of 34 IM samples from 33 patients were analyzed for the presence of *GNAS* mutations, of which 29 samples were analyzed using a gene-specific TaqMan genotyping assay for the detection of *GNAS* hotspot mutations c.601C > T and c602G > A in IM, and 32 samples using a novel next generation sequencing (NGS)-based approach employing single-molecule tagged molecular inversion probes (smMIP) to identify mutations in exon 8 and 9 of *GNAS*. Results between the two assays were compared for their ability to detect *GNAS* mutations with high confidence.

**Results:**

In total, 23 of 34 samples were successfully analyzed with both techniques showing *GNAS* mutations in 12 out of 23 (52%) samples. The remaining 11 samples were analyzed with either TaqMan assay or smMIP assay only. The TaqMan assay revealed *GNAS* mutations in 16 out of 29 samples (55%), with six samples c.601C > T (p.R201C; 38%) and ten samples c.602G > A (p.R201H; 62%) missense mutations. The smMIP assay identified mutations in 16 out of 28 samples (57%), with five samples c.601C > T (p.R201C; 31%) and seven samples c.602G > A (p.R201H; 44%) missense mutations. In addition, four samples (25%) revealed novel IM-associated mutations, including c.601C > A (p.R201S), c.602G > T (p.R201L), c.602G > C (p.R201P) and c.680A > G (p.Q227R). Combining the results of both tests, 23 out of 34 sporadic IM samples (68%) showed a *GNAS* mutation.

**Conclusions:**

Both the TaqMan and the smMIP assay a show a high degree of concordance in detecting *GNAS* hotspot mutations in IM with comparable sensitivity. However, since the NGS-based smMIP assay permits mutation detection in whole exons of *GNAS*, a broader range of *GNAS* mutations can be identified by the smMIP approach.

## Background

Intramuscular myxoma (IM) is a benign soft tissue neoplasm that belongs to the group of myxoid tumors characterized by a marked abundance of extracellular myxoid matrix. These tumors share several histological features, and depending on their clinical presentation and place of origin, can be subdivided into intramuscular, superficial-cutaneous, odontogenic and juxta-articular myxoma [1, 2]. These myxomas all represent distinct entities with different characteristic gene lesions involved in their pathogenesis. Therefore, gene mutation analysis can be very helpful in differential diagnosis to support the histopathology of these tumors [3, 4].

IM is characterized by bland spindle- and/or stellate-shaped cells embedded in a hypovascular, abundant myxoid stroma. The nuclei are small showing no or minimal nuclear atypia. Often areas with increased cellularity can be observed and when hypercellular areas predominate it is designated as cellular myxoma [2, 5, 6], which can easily be confused with low-grade fibromyxoid sarcoma or low-grade myxofibrosarcoma, especially in very small biopsies. IM is a somatic mosaic disorder generally occurring as a sporadic solitary neoplasm, although it can be part of Mazabraud’s syndrome characterized by a combination of polyostotic fibrous dysplasia with multiple IM’s [7, 8]. Mazabraud’s syndrome and the closely related McCune-Albright syndrome, which is associated with fibrous dysplasia, café au lait macules and endocrine disorders, are caused by activating missense mutations in codon 201 of the *GNAS* gene [8–12].

*GNAS* encodes the stimulatory G-alpha subunit of the heterotrimeric G-protein complex, which regulates activation of adenylyl cyclase that converts adenosine triphosphate (ATP) into cyclic adenosine monophosphate (cAMP). Overproduction of second messenger cAMP and activation of downstream signaling pathways has been observed in cells harboring *GNAS* mutations [13, 14]. In 2000, Okamoto et al. first described somatic post-zygotic *GNAS* mutations in IM with and without fibrous dysplasia [8]. Thereafter, three more studies showed that *GNAS* lesions occur frequently in sporadic IM, which were detected in 36–61% of the cases, and exclusively involved c.601C > T (p.R201C) and c.602G > A (p.R201H) mutations [8, 15, 16]. On the other hand, *GNAS* mutations are absent in low-grade myxofibrosarcoma, which can be useful in the differential diagnosis with (cellular) IM [17, 18]. Notably, juxta-articular myxoma and cardiac myxoma also lack *GNAS* driver mutations [4, 16].

A complicating factor for mutation detection in IM is the mosaicism of *GNAS* mutations combined with hypocellularity of the tumor, where low concentrations of genomic DNA are isolated from these tissue specimens, especially in the case of biopsy material. In the past decades, several techniques for *GNAS* mutation detection have been developed and used [8, 19–21]. In 2009, Delaney et al. tested 28 IM’s for *GNAS* mutations by using conventional PCR followed by mutation-specific restriction enzyme digestion (PCR-MSRED) and COLD-PCR/MSRED and showed that COLD-PCR/MSRED was more sensitive than the conventional PCR (61% vs. 29% mutations) [15]. Thus, this tumor type may benefit from the development of more robust and sensitive techniques for mutation detection, such as next generation sequencing (NGS). Recently, our molecular diagnostic laboratory has developed a novel NGS-based approach employing single-molecule molecular inversion probes (smMIP) that combines multiplex analysis with single-molecule tagging, also named Unique Molecule Identifiers (UMI) [22, 23]. By using this method, duplicate reads can be identified and merged into a single consensus, reducing false-positive calls originated during PCR and sequencing and allowing a technical sensitivity of 1% mutant allele. In addition, the actual number of sequenced genomic DNA (gDNA) molecules can be determined, which is especially relevant when analyzing limited amounts of gDNA. Furthermore, the strand-specific amplifications allows the distinction between genuine C > T and G > A mutations from deamination artifacts frequently observed when sequencing gDNA in older formalin-fixed paraffin-embedded (FFPE) tissue specimens. [22, 23].

In this study, we applied both TaqMan-based assays and the smMIP technique for *GNAS* mutation detection in IM, and compared both methods for reliable mutation detection in a diagnostic setting.

## Methods

### Patient samples

This study included 34 samples of sporadic intramuscular myxoma from 33 patients that were collected retrospectively (from 1998 till 2018) from archives of the Pathology Departments in the Netherlands of the Radboud University Medical Centre, Jeroen Bosch Hospital (Den Bosch), PAMM institute (Eindhoven) and Rijnstate Hospital (Arnhem). None of the patients were prior diagnosed with fibrous dysplasia or developed this during follow-up. From one patient, two samples (sample 28 and 29) were analyzed, which yielded identical data for mutation analyses. For each case, a 4 μm thick section of FFPE material was stained with haematotoxylin and eosin (H&E). The histological diagnoses were revised (UF, EB) and classified according to the 2013 World Health Organization criteria [2]. The samples included in this study complied with the standards of the Committee for Human Research Ethics (CMO).

### DNA isolation

Three 20 μm thick sections were cut from each specimen of FFPE tissue and were digested at 56 °C for at least 1 h in the presence of TET-lysis buffer (10 mmol/L Tris/HCl pH 8.5, 1 mmol/L EDTA pH 8.0, 0.01% Tween-20) with 5% Chelex-100 (143 to 2832; Bio-Rad, Hercules, CA), 15 μg/mL GlycoBlue (AM9516; Thermo Fisher, Waltham, MA), and 400 μg proteinase K (19,133; Qiagen, Valencia, CA), followed by inactivation at 95 °C for 10 min. DNA concentration for TaqMan assay was assessed with the NanoDrop Microvolume Spectrophotometer (Peqlab Biotechnologies, Erlangen, Germany) and for smMIP assay with the Qubit Broad Range Kit (Q32853; Thermo Fisher). To concentrate the DNA samples for the robotized protocol of the smMIP procedure, supernatant was cooled on ice and precipitated in the presence of 70% EtOH and 1/10 volume 3 M NaAc (pH 5.2). Pellets were washed with cold 70% EtOH and dissolved in 80 μL Tris-EDTA. DNA quality of the samples was tested using a size ladder control PCR, in which gene segments of house-keeping genes were amplified, yielding different fragment sizes (100, 200, 300 and 400 bp), depending on the extent of fragmentation of the DNA.

### TaqMan genotyping assay

Pre-designed and validated gene-specific TaqMan Genotyping Assays from Thermo Fisher Scientific was used for quantitative real-time RT-PCR. Every set contained gene specific forward 5’-CTTTGGTGAGATCCATTGACCTCAA-3′ and reverse primers 3’-CACCTGGAACTTGGTCTCAAAGATT-5′ and fluorescence labeled probes (Table 1). Probes are spanning an exon junction to detect genomic DNA. The PCR reaction volume was 20 μl and contained 1 μl DNA (10 ng/μl), 10 μl TaqMan Universal PCR Mastermix NoAmpErase UNG (Applied Biosystems, Foster City, CA), 0,5 μl predesigned and validated gene-specific TaqMan Gene Expression Assay mix (Applied Biosystems), 0,5 μl TE buffer (Promega) and 8 μl water. ABI Prism 7500 Real-Time PCR system (Applied Biosystem) was used to amplify codon 201 of exon 8 of the *GNAS* gene from each sample on a 96-well reaction plate with the following protocol: 10 min denaturation at 95 °C, 40 cycles of 15 s denaturation at 95 °C, 1 min annealing and extension at 60 °C. Dilution studies were performed using fibrous dysplasia samples harboring the two previously described *GNAS* mutations. The limit of detection was reliable at a variant allele frequency (VAF) of 5%.

**Table 1:** Fluorescent reporter probes for TaqMan assay

TaqMan assay	Reporter probe wild-type	Reporter probe mutant
*GNAS* c.601C > T	5’-CAGGACACGGCAGCGA-3’	5’-CAGGACACAGCAGCGA-3’
*GNAS* c.602G > A	5’-TTCGCTGCCGTGTCCT-3’	5’-CGCTGCCATGTCCT-3’

Underscored nucleotides are hot-spot mutation position

### Next generation sequencing with single-molecule molecular inversion probes

The single-molecule molecular inversion probe (smMIP) procedure was performed as described elsewhere [22]. In short, a pool of smMIPs covering 41 mutational hotspot regions of 23 distinct genes, including *GNAS*, was phopshorylated with T4 polynucleotide kinase. A total of 100 ng genomic DNA was used as input in the capture reaction with the diluted phosphorylated smMIP pool. After extension, ligation and exonuclease treatment, PCR reactions were performed with barcoded reverse primers and iProof high-fidelity master-mix (Biorad). PCR reactions of the different samples were pooled, and purified with 0.8 x volume of Agencourt Ampure XP Beads (Beckman Coulter, Brea, CA). The purified libraries were prepared for sequencing on a NextSeq 500 instrument (Illumina, San Diego, CA) according to the manufacturer’s protocol (300 cycles Mid Output sequencing kit, v2), resulting in 2 × 150 bp paired-end reads. Data analyses were performed as previously described [22]. Variants were called at a VAF of > 1% and ≥ 3 mutant gDNA molecules and a minimum of 20 gDNA molecules analyzed at that position. Samples that did not fulfil the standard settings with respect to gene coverage in combination with tumor load were marked as inconclusive [22].

## Results

### Histopathology and clinical information of intramuscular myxoma cases

**Fig. 1:**
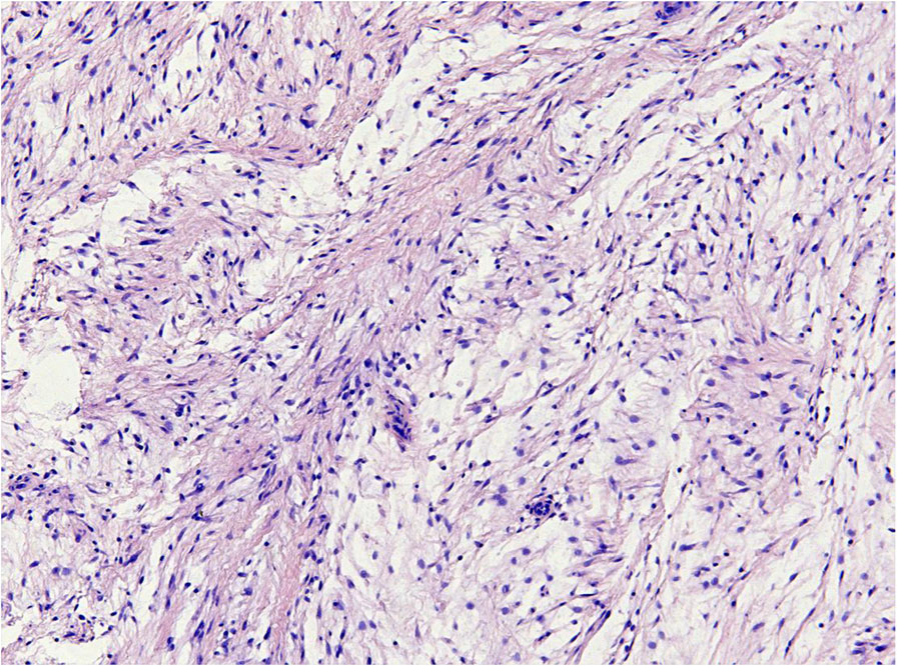
Representative photomicrograph of heamatoxylin and eosine (H&E)-stained section of a cellular intramuscular myxoma showing its characteristic histological morphology

Histopathology of H&E-stained slides confirmed that a selected set of 34 samples from 33 patients showed the classical features of IM, which were composed of uniform, sparsely distributed cytological bland spindle- or stellate-shaped cells with tapering eosinophilic cytoplasm and small nuclei embedded in an abundant myxoid stroma. One case showed prominent hypercellular areas with more collagenous stroma and was diagnosed as cellular myxoma according to the criteria defined by Nielsen et al (Fig. 1) [5, 6]. Of the 34 myxoid tumors, 31 samples were obtained by local excision and 3 samples by needle biopsy (Table 2). From one patient (sample 28 and 29), a biopsy and the following excision were analyzed for *GNAS* mutational status. No recurrences were reported in any of the cases and no additional treatment was given. Follow up time ranged between 2 months and 21 years.

**Table 2:** Patient characteristics

Patient	Age at presentation (years)	Gender	Tumor localization	Tumor size (cm) excision	Follow-up (months)
1	74	M	Lower leg	14	207
2	42	F	Thigh	5	201
3	51	F	Upper arm	0.8	128
4	45	M	Upper leg	5.5	126
5	40	F	Thigh	Bx	99
6	47	F	Lower leg	10	93
7	51	M	Shoulder	3	85
8	64	M	Thigh	5.2	83
9^a^	53	M	Upper arm	2	83
10	45	M	Thigh	5.8	82
11	57	F	Thigh	9	67
12	72	M	Thigh	3.5	63
13	71	M	Back	3	210
14	55	M	Thigh	4	182
15	55	M	Thigh	4	157
16	53	F	Upper arm	3	156
17	47	F	Upper arm	5.5	100
18	38	F	Upper arm	2	108
19	57	M	Thigh	3.2	126
20	39	F	Upper arm	7	191
21	69	F	Chest	2	63
22	46	F	Shoulder	3.5	200
23	64	F	Thigh	3	194
24	46	M	Thigh	5	181
25	67	F	Lower arm	1.7	127
26	49	F	Thigh	3	73
27	58	F	Thigh	3	244
28^b^	33	F	Thigh	7.5	224
29	65	M	Thigh	5.5	31
30	59	F	Thigh	7	12
31	40	F	Upper arm	Bx	260
32	71	F	Thigh	1	6
33	54	M	Thigh	Bx	2

Bx: biopsy, ^a^cellular myxoma, ^b^patient with two intramuscular myxoma samples

### GNAS mutation detection in intramuscular myxoma

**Fig. 2:**
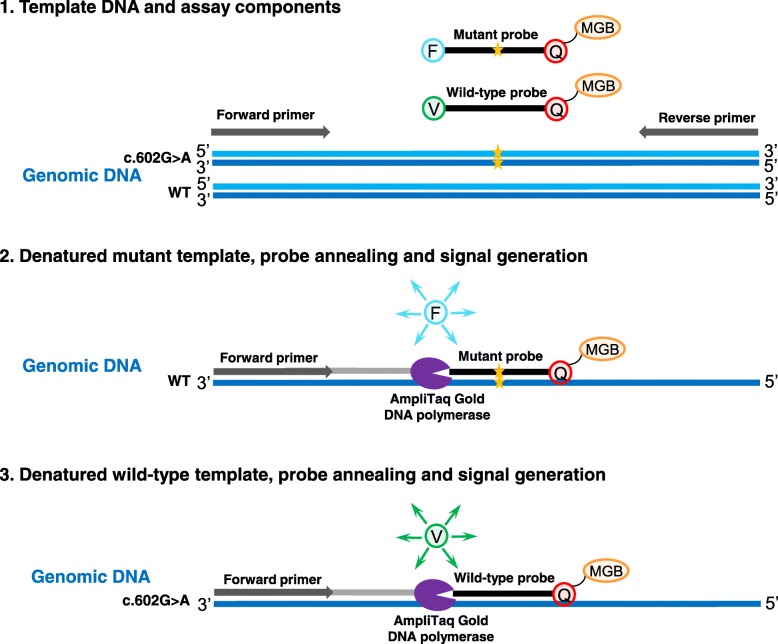
Schematic overview of the Taqman assay. In addition to the genomic DNA template, four additional oligonucleotide components are required to detect the mutation. These include an unlabeled PCR primer pair and two TaqMan probes with a FAM (F) or a VIC (V) dye label on the 5’end, in combination with a minor groove binder (MGB) and a nonfluorescent quencher (Q) on the 3’end (1). The TaqMan probes hybridize to the target DNA after denaturation between the unlabeled PCR primers. The signal from the fluorescent dye on the 5’end of a TaqMan probe is quenched by the quencher on its 3’end through fluorescence resonance energy transfer (FRET) (2). During PCR, the AmpliTaq Gold DNA polymerase extends the unlabeled primers using the genomic DNA template strand. When the DNA polymerase reaches the TaqMan probe, it cleaves the molecule, separating the fluorescent dye from the quencher. The qPCR instrument detects fluorescence from the unquenched FAM or VIC dye in one reaction (3)

All samples (*n* = 34) were tested for the presence of *GNAS* mutations, 32 samples using the smMIP assay and 29 samples with the TaqMan genotyping assay. First, *GNAS* mutation detection was performed for 29 DNA samples with the TaqMan assay, where specific fluorescently labeled probes were used for the detection of c.601C > T (p.R201C) and c.602G > A (p.R201H) hotspot mutations (Fig. 2). Each sample was analyzed in two independent assays together with both negative and positive control samples. *GNAS* genetic alterations were identified in 16 out of 29 samples (55%), with six samples c.601C > T (38%) and ten samples c.602G > A (62%) mutations (Table 3). From one patient both samples (biopsy and excision) were positive for c.601C > T mutation (Table 3; sample 28 and 29).

**Table 3:** Mutation analysis intramuscular myxoma

Sample	Taqman assay (WT/Mut/Inc)	smMIP assay (WT/Mut/Inc)	Mutation	Amino acid substitution	Mutant allele frequency (smMIP)	Concordance between Taqman and smMIP assay
1	WT	WT				Concordant
2	Mut	Mut	c.601C > T	p.R201C	5%	Concordant
3	WT	WT				Concordant
4	WT	WT				Concordant
5^a^	WT	Mut	c.680A > G	p.Q227R	27%	Mutation not included in TaqMan assay
6	WT	WT				Concordant
7	Mut	Mut	c.602G > A	p.R201H	13%	Concordant
8	Mut	Mut	c.602G > A	p.R201H	14%	Concordant
9	WT	WT				Concordant
10	Mut	Mut	c.602G > A	p.R201H	26%	Concordant
11	Mut	NA	c.601C > T	p.R201C		Not analyzed by smMIP
12	WT	WT				Concordant
13	WT	WT				Concordant
14	Mut	Mut	c.601C > T	p.R201C	13%	Concordant
15	Mut	Inc	c.602G > A	p.R201H		Insufficient quality for smMIP assay
16	WT	WT				Concordant
17	WT	WT				Concordant
18	Mut	Inc	c.602G > A	p.R201H		Insufficient quality for smMIP assay
19	Mut	Mut	c.602G > A	p.R201H	10%	Concordant
20	Mut	Mut	c.602G > A	p.R201H	9%	Concordant
21	Mut	NA	c.602G > A	p.R201H		Not analyzed by smMIP
22	Mut	Mut	c.601C > T	p.R201C	15%	Concordant
23	Mut	Mut	c.602G > A	p.R201H	19%	Concordant
24	WT	WT				Concordant
25	WT	WT				Concordant
26*	WT	Mut	c.602G > T	p.R201L	15%	Mutation not included in TaqMan assay
27	Mut	Mut	c.602G > A	p.R201H	14%	Concordant
28^b^	Mut	Inc	c.601C > T	p.R201C		Insufficient quality for smMIP assay
29^b^	Mut	Inc	c.601C > T	p.R201C		Insufficient quality for smMIP assay
30^a^	NA	Mut	c.602G > C	p.R201P	12%	Not analyzed by Taqman
31	NA	Mut	c.601C > T	p.R201C	13%	Not analyzed by Taqman
32^a^	NA	Mut	c.601C > A	p.R201S	14%	Not analyzed by Taqman
33	NA	Mut	c.601C > T	p.R201C	17%	Not analyzed by Taqman
34	NA	Mut	c.602G > A	p.R201H	7%	Not analyzed by Taqman

WT: wild-type; Mut: mutation identified; Inc.: inconclusive; NA: not analyzed

^a^Samples with novel mutations in smMIP assay which are not included in the TaqMan assay

^b^Two samples tested from the same patient (biopsy and excision)

**Fig. 3:**
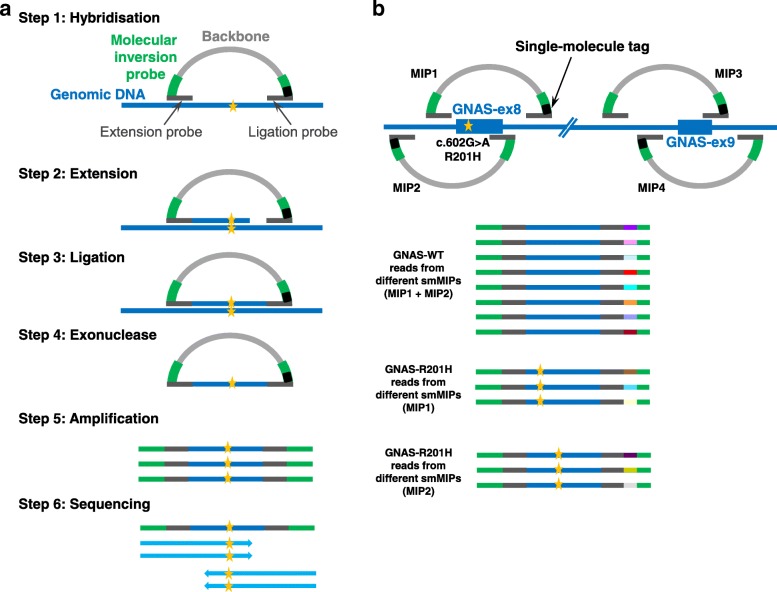
Schematic overview of the smMIP assay. (**a**) First, the single molecule molecular inversion probe (smMIP) capture procedure is performed. smMIPs are long oligonucleotides consisting of two targeting arms (extension probe and ligation probe), joined by a backbone. The probe sequences are complementary to genomic DNA sequences surrounding the target region that covers a hotspot location (indicated by the yellow asterisk). During the capture reaction, smMIPs are hybridized to genomic DNA (gDNA), followed by an extension and ligation reaction, which results in circular smMIPs. Subsequent exonuclease treatment will remove all linear gDNA and unused smMIPs. Between the backbone and probe sequences are primer sequences (green bars) that are used to amplify the target region, followed by library preparation and next-generation sequencing (NGS). (**b**) By including a single-molecule tag of 8 random nucleotides (N_8_) at the end of the ligation probe, duplicate reads can be identified and merged into a consensus thereby removing PCR and sequencing artifacts. Genuine C > T and G > A mutations can be distinguished from deamination artifacts by strand specific amplification of the smMIPs. In our smMIP design, exon 8 and exon 9 of the GNAS gene are each covered by two independent smMIPs targeting both strands (smMIP1–2 and smMIP3–4, respectively)

Next, we determined the presence of *GNAS* mutations in exon 8 and exon 9 by the smMIP assay. Within the smMIP Cancer Hotspot Panel, two smMIPs covered *GNAS* exon 8 (providing sequencing analysis of both DNA strands for a total 74 bp) and two smMIPs *GNAS* exon 9 (sequencing analysis of 59 bp), respectively (Fig. 3). Other mutational hotspot regions that were covered by smMIPs included *BRAF, CTNNB1, EGFR, HRAS, KRAS, NRAS, IDH1, IDH2* and *KIT* (for the complete list see [22]). The smMIP assay was performed on 32 samples in total, including the 27 samples that also showed a successful TaqMan assay and an additional set of 5 IM samples. From these 32 samples, the NGS data of 5 samples (for which a successful TaqMan assay was available) was based on a very limited number of gDNA molecules, and therefore could not reliably be interpreted, most likely because of very low cellularity of the IM sample and/or inferior DNA quality (Table 3; inconclusive, Inc). In total, 16 out of 28 samples (57%) showed a *GNAS* mutation, with five samples c.601C > T (31%) and seven samples c.602G > A (44%) mutations. In addition, four samples (25%) revealed novel IM-associated mutations, including c.601C > A (p.R201S), c.602G > T (p.R201L), c.602G > C (p.R201P) and c.680A > G (p.Q227R).

Combining the above, 23 samples were successfully analyzed with both techniques showing *GNAS* mutations in 12 out of 23 (52%) samples. Collectively, our data demonstrate that in 23 out of 34 IM samples (68%) a *GNAS* mutation was detected using either TaqMan and/or smMIP assay. All samples that were successfully analyzed with both approaches and harbored the classical c.601C > T or c.602G > T mutations were identified with both methods.

In total, eight samples showed the classical c.601C > T mutation (35%) and eleven samples harbored the c.602G > T mutation (48%). All hotspot mutations detected by smMIP were also identified by the TaqMan assay, including samples with a VAF of 5% (sample 2). On the other hand, due to the more stringent settings of the smMIP assay, five cases with hotspot mutations identified by TaqMan assay did not yield sufficient data by the smMIP approach for reliable interpretation. In contrast, the smMIP assay allowed the detection of four novel (potential) pathogenic *GNAS* mutations (17% of the 23 mutated samples) beyond the c.601C > T and c.602G > A mutations, not previously described for IM. Thus, both assays provide merits in the molecular diagnostics of IM.

## Discussion

Intramuscular myxoma (IM) mostly occurs sporadically in the skeletal muscle of the thigh. These lesions affect mainly middle-aged adults, women more often than man [1, 24]. The prevailing view is that driver mutations of this neoplasm are exclusively located in codon 201 of the *GNAS* gene, encoding the stimulatory G-protein alpha subunit that activates the enzyme adenylate cyclase. Due to the low cellularity and somatic mosaicism in most of these lesions, mutation detection can be quite challenging and the presence of a mutation can be easily missed.

In our study we used two different techniques (TaqMan and smMIP assay) to compare the detection sensitivity of *GNAS* mutations in these lesions. In our series, 23 out of 34 sporadic IM cases (68%) showed a *GNAS* mutation, 16 out of 29 samples (55%) in the TaqMan assay and 16 out of 28 samples (57%) in the smMIP assay of which 23 samples were successfully analyzed with both techniques showing *GNAS* mutations in 12 out of 23 (52%) samples. The test-specific detection rate was 55% with the TaqMan assay and 57% for the smMIP approach. The VAF for the TaqMan assay was determined at > 5% in this study and the required input was only 10 ng gDNA. The VAF for smMIP was set at > 1% and a minimum of 3 mutant gDNA molecules, and a coverage of 20 gDNA molecules. This demonstrates that both tests are sensitive methods and useful for molecular diagnostics of tumor samples harboring mutations with a low mutant allele frequency.

In comparison, Walther et al found *GNAS* mutations in 37% (23/63) of IMs with direct Sanger sequencing and Delaney et al detected mutations in 61% (17/28) using COLD-PCR/MSRED [15, 16]. However, the smMIP technique, because of the whole exon sequencing nature of this test, allowed detection of four additional mutations that previously have not been described in IM. By using smMIP we identified one c.680A > G mutation in exon 9, and three novel mutations in exon 8, one mutation at position c.601, namely c.601C > A, and two mutations at position c.602, which included c.602G > C and c.602G > T. The c.601C > A mutation has previously been reported in fibrous dysplasia, while the c.602G > C and c.602G > T mutations were only reported in sporadic endocrine tumors so far [9, 10, 19, 25]. These mutations were not detected by TaqMan, since this assay was designed to report only the two classical hotspot mutations c.601C > T and c.602G > T.

The smMIP approach allows the distinction between genuine C > T and G > A mutations from deamination artifacts frequently observed when sequencing gDNA from FFPE tissue specimens [22]. All cases harboring a C > T or G > A mutation in *GNAS*, mutant reads originating from both DNA strands were observed, showing that these represent genuine mutations. Since the TaqMan approach does not allow this distinction, deamination artifact could potentially cause false positive results. In our study, all hotspot mutations detected with the Taqman assay were confirmed with the smMIP technique, indicating no false-positive results with Taqman. Even samples with a VAF of around 5% could be detected by both TaqMan assay and smMIP. The four samples with a hotspot mutation that were detected by TaqMan, but did not yield a reliable smMIP assay result, all had a mutated VAF of approximately 10–30% as judged by Taqman assay, and were therefore interpreted as true mutations.

Significant benefits of the TaqMan assay include low cost and short turn-around time (≤2 working days). A limitation of Taqman is that within one assay only one or two hotspot mutations can be detected. For smMIP analysis the turn-around time in our laboratory is ≤7 working days. A large initial investment was needed and high numbers of samples are required for parallel analyses to have a cost-efficient test. Because multiple genes can be tested at once with the smMIP assay, large amounts of samples are relatively easy obtained in routine clinical setting with the current demand of molecular diagnostics [22]. Because of the sensitive characteristics of the smMIP technique and its accuracy of mutation detection on FFPE material as well as the broader coverage of the *GNAS* gene, this technique to our opinion is preferable.

The most important differential diagnoses of IM, especially the cellular variant, are low-grade fibromyxoid sarcoma and low-grade myxofibrosarcoma. In biopsy material the distinction can be challenging and in those cases molecular diagnostics can be beneficial. A specific immunohistochemical and molecular signature is well known for low-grade fibromyxoid sarcomas with expression of MUC4 and the presence of *FUS/EWSR1-CREB 3 L2/1* fusions making a distinction from IM easily possible [17, 18]. In contrast, for low-grade myxofibrosarcoma, specific immunohistochemical or molecular characteristics are lacking. Sensitive molecular tests, like smMIP and TaqMan assays for *GNAS* mutation analysis, might be very helpful in assessing the diagnosis, which has therapeutic consequences when considering malignancy [21]. Nevertheless there are also cases in which no *GNAS* mutation could be detected, suggesting that there are still other aberrations to be identified in IM.

Panagopoulos et al recently found abnormal karyotypes in 21 out of 68 cases, with nine cases showing nonrandom involvement of chromosome 8 (which harbors the *GNAS* gene) with seven cases showing trisomy 8, one with a deletion and one with a translocation. Only one case in their series showed a c.601C > T *GNAS* mutation [26]. Thus, chromosomal aberrations could be an alternative explanation for at least a subset of the non-mutated cases.

The smMIP-NGS cancer hotspot panel that was employed to check for *GNAS* mutations, also contained smMIPs that covered mutational hotspots in the genes *AKT1, BRAF, CTNNB1, EGFR, ERBB2, GNA11, GNAQ, H3F3A, H3F3B, HRAS, IDH1, IDH2, JAK2, KRAS, MPL, MYD88, NRAS, PDGFRA* and *PIK3CA.* In none of the 32 samples that could be reliably analyzed by smMIPs, additional mutations were detected in these regions. Thus, *GNAS* mutations represent a unique driver mutation for this benign tumor type.

## Conclusion

In conclusion, both TaqMan and smMIP assay are comparably sensitive molecular methods with valuable applicability in diagnostic pathology for IM. Furthermore, due to a broader coverage of the *GNAS* gene by the smMIP approach, four novel IM-associated missense mutations of *GNAS* could be identified (17% of all mutated samples), which previously have only been reported in McCune-Albright syndrome and sporadic endocrine tumors.

## Abbreviations

ATP: adenosine triphosphate
cAMP: cyclic adenosine monophosphate
CMO: Committee for human research ethics
FFPE: formalin-fixed paraffin-embedded
FRET: fluorescence resonance energy transfer
H&E: haematotoxylin and eosin
IM: intramuscular myxoma
MGB: minor groove binder
NGS: next generation sequencing
smMIP: single-molecule molecular inversion probes
UMI: Unique molecule identifiers
VAF: variant allele frequency
